# Effects of Endocrine-Disrupting Chemicals on Endometrial Receptivity and Embryo Implantation: A Systematic Review of 34 Mouse Model Studies

**DOI:** 10.3390/ijerph18136840

**Published:** 2021-06-25

**Authors:** Donatella Caserta, Flavia Costanzi, Maria Paola De Marco, Luisa Di Benedetto, Eleonora Matteucci, Chiara Assorgi, Maria Clara Pacilli, Aris Raad Besharat, Filippo Bellati, Ilary Ruscito

**Affiliations:** 1 Gynecology Division, Department of Medical and Surgical Sciences and Translational Medicine, Sant’Andrea University Hospital, Sapienza University of Rome, Via di Grottarossa 1035, 00189 Rome, Italy; costanzi.flavia@gmail.com (F.C.); demarco.mariapaola@gmail.com (M.P.D.M.); luisadibenedetto@outlook.it (L.D.B.); eleonora.matteucci@uniroma1.it (E.M.); chiara.assorgi@hotmail.it (C.A.); mariaclarapacilli@gmail.com (M.C.P.); besharataris@gmail.com (A.R.B.); filippo.bellati@uniroma1.it (F.B.); ilary.ruscito@uniroma1.it (I.R.); 2 PhD Program in “Translational Medicine and Oncology”, Department of Medical and Surgical Sciences and Translational Medicine, Psychology and Medicine Faculty, Sapienza University of Rome, Via di Grottarossa 1035, 00189 Rome, Italy

**Keywords:** endocrine disrupting chemicals, infertility, implantation failure, post-implantation loss, environmental pollutants, phthalate

## Abstract

Several available studies have already analyzed the systemic effects of endocrine-disrupting chemicals (EDCs) on fertile woman and neonatal outcomes, but little is still known in humans about the precise mechanisms of interference of these compounds with the endometrial receptivity. There is consistent evidence that continuous and prolonged exposure to EDCs is a risk factor for reduced fertility and fecundity in women. Preliminary studies on mammalian models provide robust evidence about this issue and could help gynecologists worldwide to prevent long term injury caused by EDCs on human fertility. In this systematic review, we aimed to systematically summarize all available data about EDC effects on blastocyst endometrial implantation. We performed a systematic review using PubMed^®^/MEDLINE^®^ to summarize all in vivo studies, carried out on mice models, analyzing the molecular consequences of the prolonged exposure of EDC on the implantation process. 34 studies carried out on mouse models were included. Primary effects of EDC were a reduction of the number of implantation sites and pregnancy rates, particularly after BPA and phthalate exposure. Furthermore, the endometrial expression of estrogen (ER) and progesterone receptors (PR), as well as their activation pathways, is compromised after EDC exposure. Finally, the expression of the primary endometrial markers of receptivity (such as MUC1, HOXA10, Inn and E-cadherin) after EDC contact was analyzed. In conclusion EDC deeply affect blastocyst implantation in mouse model. Several players of the implantation mechanism are strongly influenced by the exposure to different categories of EDC.

## 1. Introduction

**Figure 1:**
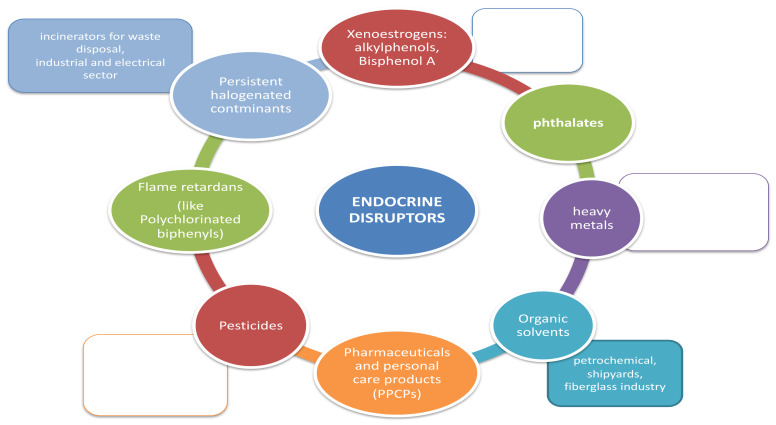
Environmental distribution of endocrine-disruptor chemicals (EDCs).

Infertility is globally recognized as a public health problem [1,2]. The World Health Organisation (WHO) defines infertility as “a disease of the reproductive system defined by the failure to achieve a clinical pregnancy after at least 12 months of regular unprotected sexual intercourse” [3]. Infertility affects 15% of couples worldwide [4] and it is defined as “primary” infertility if the couple never conceived, while it is considered as “secondary” infertility if it occurs after one or more previous pregnancies. In more than 43% of cases, the aetiology of infertility is of female origin, while in 34% of the case it is due by male factors. In 17% of cases both female and male defects contribute to the infertility of the couple, while in 10% of cases the cause of infertility remains unknown [5]. The implantation failure remains an unsolved problem in reproductive medicine and is considered as a consistent cause of unexplained infertility in healthy women. It has been estimated that 75% of pregnancy losses are associated with implantation failure [6]. It is generally accepted that embryo implantation depends on both blastocyst and endometrial quality and on the synchronization of their development [7,8]. The endometrial ability to support embryo implantation is called “endometrial receptivity”. This ability occurs exclusively at a specific period in the menstrual cycle termed “implant window” that generally corresponds, in women, to the mid-luteal phase [9]. During the luteal phase, indeed, the decidualization process occurs where the increase of progesterone induces the endometrial stromal cells differentiation into the largest decidual cells. Therefore, a functional consequence of decidualization is that uterus becomes transiently receptive to the embryo’s implantation. Several molecular mechanisms are involved in the implantation and decidualization process [10,11]. Two hormones, estrogen and progesterone, are mainly involved in the implantation process and act on endometrial receptivity through two receptors: the estrogen receptor (ER) and the progesterone receptor (PR) [12]. Both ER and PR allow the regulation of different fundamental molecules in the decidualization and embryo implantation processes, such as homeobox transcription factors, cytokines, cyclooxygenase derived Prostaglandins and growth factors. The most involved molecules in the implantation process [13] are leukaemia inhibitory factor (LIF) [14], homeobox A10 (HOXA10), and adhesion molecules such as mucin 1 (MUC1) [15,16,17,18,19]. Increasing evidence indicates that exposure to environmental contaminants negatively affects animal and human health. These chemicals are present in various products of daily use [20] (Figure 1). They have been defined by WHO as “an exogenous substance or mixture that alters function[s] of the endocrine system and consequently causes adverse health effects in an intact organism, or its progeny, or [sub] populations” [21]. The homeostasis of the thyroid and sex steroids are the main targets of endocrine-disruptor chemicals (EDCs). Therefore, reproductive health is recognized as being especially vulnerable to EDC [22,23,24]. Materno-fetal transmission of EDC with negative impact on fetal outcome has also been demonstrated [25]. EDC are a heterogeneous group of substances of different use, chemical structure and mechanism of action. Studies on animal models suggest that exposure to EDC may play a role in the pathogenesis of infertility [24,26]. Previous data had also investigated the association between exposure to EDC and human infertility: they have shown that prolonged exposures to EDC can cause different reproductive disorders, including precocious puberty, oocyte aneuploidy, as well as an alteration in reproductive efficiency [27,28,29,30,31,32] (Table 1).

To date, there is a strong need to understand the precise molecular mechanisms involved in infertility and altered by EDC. In particular, there is a strong need to explain unexplained infertility in terms of endometrial receptivity and influence of EDC. At this scope, with the present work we aimed to systematically summarize all available data about EDC effects on blastocyst endometrial implantation process, in order to give new insight, which could help gynecologists worldwide to prevent long-term injuries caused by EDCs on human fertility.

## 2. Materials and Methods

**Figure 2:**
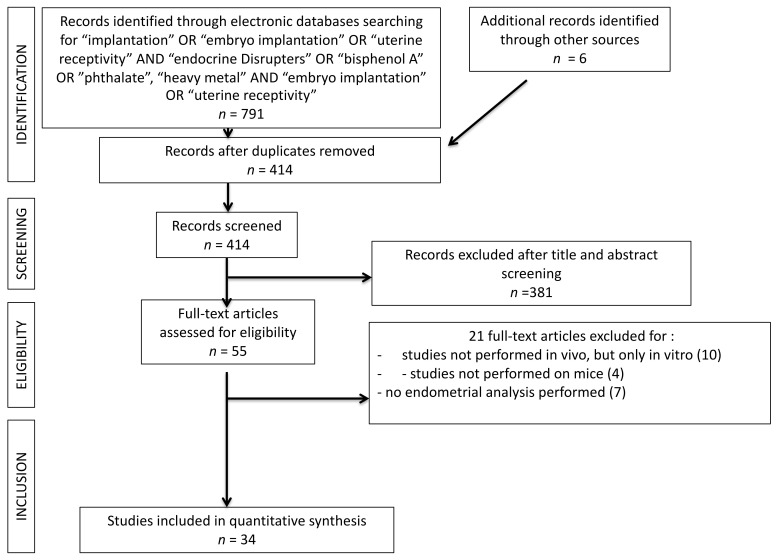
PRISMA flow chart. 381 records were excluded after title and abstract screening because, basing on inclusion and exclusion criteria, they were not pertinent with the searched studies.

The present systematic review was carried out following the Preferred Reporting Items for Systematic Reviews and Meta-Analyses (PRISMA) guidelines. All in vivo studies analyzing the interaction of EDC on the blastocyst implantation process were searched. A search was performed on the PubMed^®^/MEDLINE^®^ database and restricted for the last 20 years of publication (2000–2020). Only studies in English were included. The search was carried out in October 2020. The terms used for the search were: (“Endocrine Disrupters” OR “Heavy Metals”, OR “Bisphenol A” OR “Phthalates”) AND (“Embryo Implantation” OR “Implantation” OR “Uterine Receptivity”). A total of 791 results were obtained. One hundred fourteen duplicates were excluded. A total of 381 articles were excluded by reading the abstract and title. Fifty-five articles remained for reading the full texts. Among these studies, a total of 21 studies were excluded: 11 studies were excluded because they were about in vitro experiments, four studies because they investigated other mammalian species than mouse models; six studies because an endometrial receptivity analysis was not performed. A total of thirty-four articles were finally included into the systematic review. The study selection process was summarized on PRISMA flow chart (Figure 2).

## 3. Results

### 3.1. EDC Effects on Embryo Implantation

#### 3.1.1. Bisphenol A (BPA)

BPA is an aromatic compound that is a precursor of plastic materials and chemical additives; it is used in the production of polycarbonate plastics (very popular due to the thermic properties, transparency, and mechanical resistance) and for the production of containers for food use or in epoxy resins (internal protective coating of most food and beverage receptacles). BPA is one of several chemicals that potentially interact with the body’s hormonal systems. It has been known that BPA can mimic female sex hormones, especially estrogen. The effects of BPA on fertility, reproduction and the endocrine system are the subject of many scientific studies.

The analysis of the association of BPA with the pregnancy rate and the number of implantation sites in murine studies were analyzed in 16 available papers [33,34,35,36,37,38,39,40,41,42,43,44,45,46,47]. These studies differentiated for exposure dosage and administration period. Eleven papers [33,34,35,36,38,40,41,42,44,45,47] analyzed the effects of BPA administration, given orally or subcutaneously, during the pre-implantation period. Results obtained show that the number of implantation sites appears significantly reduced in most studies and that this number was inversely correlated with the administered BPA dosage dose [34,36,38,40,41,45,47]. Two studies did not find a reduction in the number of implantation sites after BPA exposure [33,35]. A more complex analysis was carried out by the study group of Borman et al. [42], which demonstrated that the association of a stressful condition, together with a BPA dosage higher than 4 mg leads to a reduction in implantation sites. The same result was not observed in mice subjected only to stress conditions or only administered with BPA. Crawford et al. [44] analyzed the effect of triclosan, an antimicrobial agent used in various consumer products, individually and in combination with BPA, differentiating the effects on the number of implantation sites with chronic or acute administration. An administration of high daily dosages (18 mg/animal/day; 27 mg/animal/day) or in a single dose in the first three days of gestation determines a decrease of the implantation sites. Furthermore, the combination of 4 mg BPA with 9 mg triclosan causes a significant decrease in implantation sites more than single administrations (*p* = 0.05).

Four other studies [28,37,39,46] investigated the effects of the BPA exposure on neonatal, prepubertal and adult mice, showing a successive reduction in implantation sites during their reproductive age. Li et al. [37] demonstrated that in prepubertal mice chronically exposed to BPA, a decrease in implantation sites is directly proportional to the administered dose and determines an alteration of the decidualization process of the stromal cells. Martinez Penaa et al. [43] also confirmed that intrauterine exposure to BPA of the offspring resulted in decreased implantation sites during their adulthood.

#### 3.1.2. Phthalate

Three studies [48,49,50] analyzed the effects of different phthalate molecules on implantation sites. Ema et al. focused their attention on the study of phthalates by analyzing two different molecules: Dibutyl phthalate (DBP) and monobutyl phthalate (MBP). A first study demonstrated how the administration of DBP determines both an increase in the incidence of pre-implantation losses with doses of 1250 and 1500 mg/kg and an increase in the incidence of pregnancy losses at doses of 750 mg/kg [49]. In a second study, they [50] confirmed the negative effects of phthalates on the implantation process by demonstrating an increased incidence of pre-implantation and post-implantation losses with MBP dosages of 1000 mg/kg. The group of Li et al. [48], indeed, focused their attention on the effects caused by bis (2-ethylhexyl) phthalate (DEHP), thus highlighting a reduction in implantation sites compared to the control group in a direct proportion to the increasing dosage of DEHP. In particular, a significant decrease in implantation sites was highlighted at DEHP dosages of 1000 mg/kg/day (*p* = 0.005) compared to the control group.

#### 3.1.3. Other EDC

Two studies analyzed the effects of phenols exposure on the implantation processes in mice. Tran et al. [33] demonstrated that a significant reduction of implantation sites occurred in the group treated with 4-*tert*-octylphenol (OP; 4-(1,1,3,3-tetramethylbutyl)phenol) (OP). Results were confirmed by the group of Qu et al. [51], which demonstrated how the number of pregnancies in mice treated with 2,30,4,40,5-pentachlorobiphenyl (PCB 118) was lower than in the control group. In four of the 12 fertilized mice no implants were seen in both groups treated with PCB 118 (1 mg/kg/d PCB 118 (*p* = 0.047) and 100 mg/kg/d PCB 118 groups (*p* = 0.047).

Six studies analyze other different endocrine disruptors [52,53,54,55,56,57]. The use of benzo(a)- pyrene (BaP) led to a significant decrease of implantation sites in the study groups treated with doses of 0.2 and 20 mg/kg. (*p* = 0.006 and *p* = 0.003, respectively). No differences from the control group were detected in mice treated with 2 mg/kg [52]. Milesi et al. [53] instead studied the effect of exposure in the first postnatal week of endosulfan. The results show a reduced pregnancy rate in adulthood with a non-pregnancy rate in 23% of cases in the endosulfan group, compared to 100% pregnancies in the control group.

The analysis of fungicides effects on implantation process was analyzed by two studies; the study chemicals were mancozeb [56] and azole fungicides [57], respectively. Both studies indicated a loss of implantation sites both in the pre-implantation and post-implantation phases. Furthermore, in accordance with the results observed with other substances, the analysis of the insecticide β-cypermethrin (β-CPR) [54] and the antimicrobial triclocarban (TCC) [55] showed a reduction of the implantation sites and pre-implantation losses, respectively. Therefore, results showed that the different EDC significantly interfere with embryo implantation; the decrease of the implantation sites, indeed, is related to the increase in dosage and administration time of the different EDC.

### 3.2. Action of EDC on Estrogen and Progesterone Receptors

Uterine preparation for embryo implantation and pregnancy maintenance involves both ovarian estrogen (E2) and progesterone (P4) [58]. Their synchronized effects on uterine structure and function enable the blastocyst to attach and initiate the implantation. Embryonic E2 is considered essential for embryo implantation in pigs, guinea pig, rabbits and hamster [12]; nuclear receptors of E2 and P4 acts on uterus through E2 receptor alpha (ERa), beta (ERb), and P4 receptor B (PRb), respectively. In mice, uterine receptivity and embryo implantation are regulated by ERa and PRb [59].

Infertility is associated with a lack of both ER and PR-B that affects ovary and uterine function. EDs, being synthetic compounds, mimic natural estrogens so they can bind to nuclear ERa.

Eleven studies were identified concerning how the expression of endometrial estrogen and progesterone receptors varies with exposure to different EDC [27,28,33,37,38,48,51,53,60,61,62,63]. Among these, five studies evaluated the effects of BPA [27,28,33,37,38,60]; two studies evaluated endosulfan [53,61] and four other studies evaluated DEHP [48], PCB [51], BaP [52] and cadmium (Cd) [63].

#### 3.2.1. BPA

In Tran et al. [33], the ERa, PRa, and PRb gene expression levels were measured by real-time PCR and normalized to that of 18S ribosomal RNA (RN18S) after BPA-exposure (100 mg/kg/day) during the preimplantation period. The uterine tissue from sacrificed mice was then analyzed. Endometrial mRNA levels of ER were not changed by BPA, while PR mRNA levels were significantly decreased. In particular, PRb mRNA levels were markedly reduced. Li et al. [37] in 2016 studied mice on the 21st day of birth exposed to 60 microg/kg/d, which is close to the reference safety dose (50 microg/kg/d) for daily human consumption [64] of BPA. They found no significant difference in ER expression in uterine tissues, while BPA exposure was associated with downregulation of PR and HAND2 expression in the uterine stroma.

In Varayoud et al. [28], the ER and PR mRNA levels were found to be lower in a BPA- exposed rat group. In neonatal rats exposed to BPA, both receptors had lower expression, especially in the subepithelial stroma in a high dose of 20 mg/kg/die.

BPA altered uterine PR expression in mice administered subcutaneously with 40 to 100 mg/kg/day BPA from gestational days 0.5 to 3.5 [38]. Bosquiazzo et al. [27] evaluated expression of the subepithelial immunohistochemical progesterone and no difference was found after exposure to a maximum dose of 20 mg/kg per day in newborn female rats. The above studies considered all mRNA levels of PR and ER, and only Bosquiazzo et al. evaluated PR and ER through immunohistochemistry.

#### 3.2.2. Endosulfan

The endometrium becomes receptive during implantation, thanks to the expression of key genes regulated by ER [60]. In Milesi et al. [53], neonatal exposure to endosulfan increased the loss of pre-implanted embryos, reducing fertility with a decrease in pregnancy rate. The uterine stromal cell proliferation defect was associated with the disruption of an endocrine pathway regulated by progesterone: the progesterone/coregulator receptor/HOXA10. Successively, Milesi’s group [61] showed that endosulfan, by hypomethylating CPG islands of DNA promoters, alters the expression of ERa in exposed neonatal rats. This study showed an increase in ERa expression and its transcription variants, ERa-OS, ERa-O, ERa-OT and ERa-E1. The role of ER was clarified by the study of Pawar et al. [62] in which ERa knockout mice, during early pregnancy, had alternated differentiation of stromal cells through a paracrine mechanism. Endometrial dysfunction is therefore caused by an alteration of the epithelium-stroma paracrine dialogue, in which ERa is the protagonist. Exposure to different doses of endosulfan caused several effects: 6 μg/kg/d increased ERa mRNA, but not the ERa protein, while with 600 μg/kg/day, both mRNA and protein expression increased. It was hypothesized that the activity of the ubiquitin-proteasome pathway was altered.

#### 3.2.3. Other EDC

Pregnant mice exposed to DEHP at 1000 mg/kg/day showed an alteration of ER, especially in the luminal epithelium, while PR was defective in stromal cells [47]. This action appears to be mediated by MAPK and NF-kB with adverse effects on female reproduction with reduced endometrial receptivity. The cause of this event was both an unbalance of formation and development of pinopods and an alteration in angiogenesis. Pinopods are finger-like protrusions that the endometrium exhibits on the surface during implantation to promote the adhesion of the embryo. The morphology of pinopods rather than the presence or absence of pinopods is considered of great importance for embryo implantation. It was observed in the study of Qu et al. [51] that PCB 118 determined poorly developed pinopods with dense microvilli on luminal epithelial cell surfaces. PCB 118 compromised the endometrial receptivity of exposed female mice; in this case a deregulation of ER expression was identified. Immunohistochemistry then demonstrated a protein alteration of the estrogen receptor, mainly in luminary and glandular epithelium, for high levels PCB exposure (100 mg/kg/d). Adult females were exposed to BaP dissolved in corn oil and administered every day by oral gavage at 0.1 mL/10 g of body weight from D1 to D5. The PCR analysis, Western blot and immunohistochemistry showed significant up-regulation of ERa and downregulation of PR in exposed mice [52].

#### 3.2.4. Heavy Metals

Cd, finally, caused a decrease in ERa immunoreactivity in both groups exposed to the C/PaPd compared to controls, in female BALB/c mice exposed to 200 ppm Cd in their drinking water for either 30 or 60 days. In particular, the decrease was more evident in the 30-day Cd group [63]. Therefore, the EDC interaction with the expression levels of the hormonal receptors underlies the main dysfunctional mechanisms in the implantation process.

### 3.3. Endometrial Receptivity Markers

As already mentioned, the implantation process is mediated by a hormonal process mainly directed by estrogen and progesterone. The hormonal interaction with their receptors activates different molecular pathways that allow the activation of endometrial receptivity markers during the implantation process. Therefore, we summarized the effects of EDC on the expression of endometrial receptivity biomarkers.

#### 3.3.1. HOXA 10 and LIF

Homeobox 10 (HOXA 10) and leukemia inhibitory factor (LIF) represent two significant biomarkers of embryo implantation. HOXA 10 is a protein encoded by the HOXA 10 gene that regulates stromal cells at the endometrial level and acts in the decidualization process. LIF, a pleiotropic cytokine of the interleukin-(IL-) 6 family, plays a key role in the endometrial transformation to receptive state, decidualization and implantation. Several studies have analyzed the effect of the use of EDC on their expression.

The effects of BPA were evaluated in three studies. Li et al. firstly demonstrated the association between BPA administration and the reduction of PR expression at the stromal level. They also demonstrated the reduction of PR stromal target expressions such as Hand2 and HOXA 10. Nevertheless, no alteration was found in the PR targets at an epithelial level such as Ihh, Alox15, and Irg1 [37]. These results were also confirmed by Varayoud et al., who demonstrated a reduced expression of the HOXA 10 genes in mice treated with BPA, especially at the subepithelial level. In a second analysis, they also analyzed the effects of BPA on the downstream targets of HOXA 10, by analyzing the levels of ITGB3 and EMX-2. The results showed an increased expression of ITGB3 and a reduced expression of EMX-2 compared to the control group [28]. Contradictory results were obtained by Tran et al., who evaluated the expression of HOXA 10 and LIF after the administration of BPA and OP. A marked reduction of HOXA 10 expression was found in the OP group at the implantation site. However, no significant reduction of HOXA 10 was present in the BPA-administered group. Regarding the expression of LIF at the implant site level, it was markedly reduced in the study groups (OP—70% and BPA—80%). [33]. LIF expression alteration, however, was not confirmed by Milesi et al. [61], who analyzed the effects of endosulfan administration in their study. Two studies also analyzed the effects of BaP on HOXA 10. [52,65] Both studies found a downregulation of HOXA 10 protein and mRNA levels, with a reduced expression at both epithelial and stromal levels. An interesting finding by Zhao et al. showed discrepancy between the HOXA 10 protein and mRNA levels at specific BaP dosages. This discrepancy, already highlighted in a previous study [29], was hypothesized to be due to the complex post-transcription regulatory mechanisms.

The effects of other EDC on HOXA 10 were analyzed in several trials. The effect of phenols was investigated by a study that analyzed exposure to PCB118 and showed a reduced expression of HOXA 10 and ITGB3 at the endometrial tissue level, mainly at the stromal level. The methylation status of HOXA 10 was also evaluated in the same study. In mice treated with PCB118 at dosages of 1 mg/kg/day and 100 mg/kg/d PCB 118, HOXA 10 was hypermethylated at 10.5% and 13% respectively, significantly higher than the control group (3.6%) (*p* = 0.031 and *p* = 0.026, respectively) [51].

The action of phthalates has been analyzed by Li et al. [48] who showed, in mice treated with DEHP, no change in the expression of the endometrial implant markers HOXA 10 and MMp-2. An altered expression of HOXA10 has also been studied with conflicting results depending on the type of EDC used. A decreased expression of HOXA10 was found following the administration of CP [54] and endosulfan [53]; on the contrary, a marked increase compared to the control group was found following contamination with CYP [66].

#### 3.3.2. Mucin 1

Mucin1 (MUC1) is a high molecular weight transmembrane proteoglycan that plays a pivotal role during the implantation process, through its downregulation during the transition from pre-receptive to the receptive phase of the endometrium, thus allowing the correct implantation process of the blastocyst. [67]. Three studies analyzed the effects caused by EDC on the expression of MUC1 in the implantation phase. Two studies focused their attention on BPA. The first study analyzed the negative effect determined by the administration of BPA and PO, by showing the increased in endometrial MUC1 expression [greater than 245% compared to the control group] [33]. Li et al. confirmed these results with the analysis of ERa targets. The study shows that although ERa expression did not changed in the mice administered with BPA, its targets underwent to significant changes: the luminal target MUC1 was significantly increased, while the stromal targets (LIF, Fra-1 and Gja) were downregulated compared to the control group [36]. Finally, Milesi et al. evaluated the effects caused by endosulfan by finding an increased expression of endometrial MUC1 and IGF-1 mRNA [61].

#### 3.3.3. E-Cadherin

E-cadherin is a cell adhesion molecule involved in the adhesion of blastocysts to the uterine wall. The immunohistochemical expression of E-cadherin is present in the apical and lateral membrane of endometrial cells, as well as on blastocyst. Martínez-Peña et al. [43] recently showed that the exposure to BPA leads to a reduction in its expression, especially at the endometrial apical membrane level.

The decrease in E-cadherin expression in both endometrial cells and blastocysts in exposed group caused a reduction in the adhesion to the uterine walls, leading to a significant reduction in the number of implant sites.

It has also been shown that E-cadherin mediated cellular adhesion regulates blastocyst compaction and internal cell mass formation. 40% of blastocysts in the BPA-exposed group have altered morphology.

Since progesterone levels also mediate the expression of cadherins, it is not known whether the influence of BPA passes through this mechanism. The same decrease in expression was observed by Borman’s 2015 [42] study in female rats exposed to BPA.

In the normal epithelial-mesenchymal transition, there is a reduction in ERa and E-cadherin expression that promotes the motility of epithelial cells and leads to cover the embryo for implantation [68]. It has been observed that there is a high expression of E-cadherin in mice groups exposed BaP 0.2 mg/kg/day. The up-regulation of E-cadherin expression interrupted the epithelial-mesenchymal transition function, which led to reduced endometrial receptivity [52].

Finally, it has been showed that DEHP downregulated the expression levels of ER and E-cadherin and those of p-ERK and NF-B with consequent alteration of the receptivity and impairment of embryonic implantation in pregnant mice receiving DEHP until 1000 mg/kg/day from day 1 of gestation until sacrifice.

#### 3.3.4. Adhesion Molecules

One study showed that BPA exposure during the perinatal period was related to alterations in TJ proteins (occludin) and FA proteins (talin) on uterine endometrial cells (UEC) [43]. Occludin (65 KDa) is expressed in the apical region of the UEC on gestational days 6 and 7 and decreased (MM 50 KDa) in the BPA administered mice compared to the control group. Talin is located in the apical and basal region of UEC during gestational day 6. It has been observed that control groups had higher talin ratios than those treated with the BPA.

One study analyzed the exposure to BPA during the early postnatal period, which modifies the expression of homeobox A10, normally expressed in the developing genito-urinary tract [40]. Beta3-Integrin (ITGB3) is a HOXA 10 target gene and is normally up-regulated. ITGB3 has been suggested to be a linking molecule between endometrium and trophoblast, as a primary connection between maternal and fetal tissues. ITGB3 expression coincides with high endometrial Hoxa10 levels in the mid secretory menstrual phase. The uterine tissue obtained from neonatally BPA20 or DES.2-exposed rats showed very low levels of ITGB3 and critical alteration of uterine functions. As a result, all constituents of the HOXA 10 pathway were affected, producing a lower number of implantation sites.

#### 3.3.5. VEGF and Co-Receptors

Vascular endothelial growth factor (VEGF) is considered one of the main regulators of uterine vasculature during the peri-implantation period. Xenoestrogen exposure (as BPA) during neonatal development, could modify regulation of VEGF and influence fertility during adult life. VEGF expression was generally increased in response to E2 (induction of ESR1) and P4 in the luminal uterine epithelium. Bosquiazzo et al. [27] analysed how the expression of VEGF is reduced after the induction of xenoestrogens. Bredhult et al. [69] observed that exposed mice, treated with 0.01, 1, or 100 µM BPA, showed decreased endothelial proliferation.

Kazi et al. [70] proved that E2, and its receptor (ESR1), normally stimulate VEGF expression in the rat uterus, but BPA exposed rats showed reduced levels of ESR1 in the uterine subepithelial stroma.

One study [27] described how neonatal exposure to BPA affected NCOR1 (a steroid receptor coregulatory in the subepithelial uterine compartment) expression in response to E2, showing an upregulation of this co-receptor. In addition, this study observed that the unusual overexpression of NCOR1 occurred in the stromal cells where VEGF induction failed, thus suggesting that high levels of NCOR1 could inhibit steroid-dependent genes in xenoestrogen-exposed animals. Furthermore, immunofluorescence results demonstrated that NCOR1 and ESR1 were present in the same uterine cells.

## 4. Discussion

**Figure 3:**
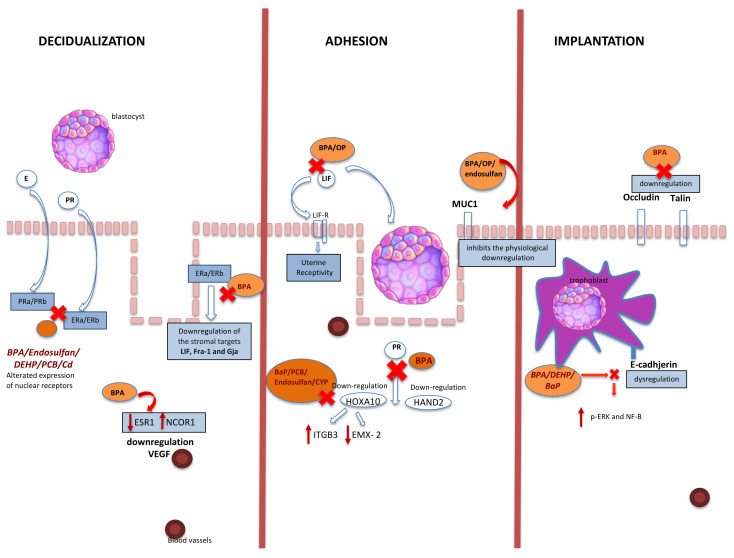
Association between EDC and embryo implantation. BPA: bisphenol A; PR: progesterone; E: estrogen; PRa/b: progesterone receptor; ERa/ERb: estrogen receptors: EP: epithelial cell (luminal and glandular epithelia); HOXA10: homeobox 10; Hand2: heart- and neural crest derivatives-expressed protein 2; LIF: leukemia inhibitory factor; LIFR: LIF receptor; DEHP: di-(2-ethylhexyl)-phthalate; VEGF: vascular endothelial growth factor; MUC1: mucin1; DEHP: di-(2-ethylhexyl) phthalate; PCB: 2,30,4,40,5-pentachlorobiphenyl; Cd: cadmium; ITGB3: integrin subunit beta 3; EMX-2: empty spiracles homeobox 2; OP: 4-*tert*-octylphenol; BaP: benzo(a)pyrene; CYP: cypermethrin; CP: β-cypermethrin; p-ERK: phosphorylation extracellular signal-related kinase NF-KB: nuclear factor-KB; NCOR1: nuclear receptor corepressor 1; ESR1: estrogen receptor 1.

The action of EDC on implantation mechanisms and endometrial receptivity were analyzed in this systematic review. Scientific evidence has repeatedly analyzed the systemic effects of EDC on fertility. Our research aimed to summarize the local molecular effects at the implant site level to understand exactly the EDC mechanisms. Endometrial receptivity requires complex interactions between the different endometrial component, including stroma, luminal and glandular epithelium, coordinated by an extended spectrum of regulatory molecules and signalling pathways [71]. Several molecular mechanisms are involved in the implantation and decidualization process. The implantation process is a limited-time phenomenon being the blastocyst phase related to the receptive status of endometrium. As a consequence, a perfect functional synchronism is essential for a successful implantation. The term “decidualization” refers to the specific endometrial and stromal transformation necessary to determine the correct process of pregnancy [72]. In mice the stimulus for decidualization, usually, is the blastocyst. In humans, instead, the initiation of this process (pre-decidualization) does not require the presence of a blastocyst but becomes definitive with implantation. The importance of pre-decidualization is to prepare the endometrium for implantation and appear equivalent to expanded stromal cell proliferation with the expression of decidual marker genes before implantation in mice [73]. Despite the number of studies that have examined potential EDC effects on female fertility in the past years [74,75], it is still difficult to layout the exact mechanism of EDC action, especially in humans, due to the difficult experimental setting. Therefore, we have reviewed the available literature on the association between EDC and embryo implantation (Figure 3) in animal models. The EDC effects reviewed were especially those ones related to the implantation sites, hormone receptors at the endometrial level, and their activation pathways (Table 2).

Ehrlich et al. [76], in a study carried out on 137 women, undergoing 180 in vitro fertilization (IVF) cycles, demonstrated a suggestive relationship between elevated urinary BPA levels and implantation failure. The molecular mechanisms underlying these findings have been demonstrated in several studies. The study of implantation sites and the pregnancy rate is analyzed in several murine studies. In the various studies analyzed, different administration techniques were used (mainly subcutaneous and oral exposure) at different times, and the effects obtained differ from the type of interferent used. The results of heterogenicity are primarily associated with the different dosages used in the studies, the administration times, and the different administration period. It is interesting to see that most of the studies focused on EDC administration in the pre-pregnancy period. Other studies also performed the analysis, in chronic or acute administration, in the perinatal period or even in the prenatal period. Despite the different protocols used, the most commonly extrapolated data is related to the association between the decrease in implantation sites and increasing dosage of interferents.

In our previous study [77] we showed a higher blood level of BPA in infertile patients and a significant increase in various nuclear receptors such as ERa and ERb. These blood markers are an indirect sign of hormonal homeostasis confirmed at the molecular level. Several studies confirmed that EDC administration influences the endometrial expression mainly of the ER and PR. It has been shown that the administration of EDC as BPA, [27,28,33,37,38,60], endosulfan [53,61], DEHP [48], PCB [51], BaP [52] and Cd [63] clearly altered their expressions. The endometrial decidualization process, together with the morphological and biochemical modifications of the endometrial stromal tissue, which is necessary for the implantation of the embryo, is a complex interaction of transcription factors, morphogens, cytokines, cell cycle regulators and signalling pathways. HOXA 10, a member of the homeobox gene family, plays a fundamental role in the decidualization and implantation process by regulating the proliferation and differentiation of endometrial stromal cells. [78]. An increased expression of HOXA 10 is found during the implantation period. The impact of EDC on HOXA 10 expression has been investigated in several studies. The role of BPA on the expression of HOXA 10 has been demonstrated by different studies [28,33,37].

In particular, Li et al. [37] demonstrated how the administration of a dosage almost similar to the permitted limits of 50 μg/kg of BPA [60 μg/kg] could interfere with the expression of PR stromal targets such as HAND2 and Hoxa10. Contrasting findings, however, were obtained by Tran et al. [33]. These data demonstrate the need for further intense monitoring of BPA exposure, even below the permitted doses. The limits of this systematic review are due to the heterogeneity of the included studies, especially regarding the administration protocol: particularly the dosage of the different EDC, the duration of administration and the period of mouse lifespan considered. In the different studies analyzed, however, an impact of EDC was found on the reduced number of implantation sites. As extensively discussed, altering a single element of the implantation process can interfere with embryo homing within the uterus. On the other hand, the strength points of the present study is the systematic nature of the review and the inclusion of all eligible studies carried out on mouse model, thus potentially eviscerating the molecular mechanisms of the EDC effects on embryo implantation thanks to the nature of experimental designs in preclinical settings.

## 5. Conclusions

In summary, a substantial body of evidence points out the need to clinically consider EDC exposure to prevent adverse effects on female fertility and fecundity, but further studies are needed to better understand the molecular mechanisms of EDC action on human females fertility. Therefore, the different EDC interfere in a heterogeneous way in the implantation mechanisms. There was a significant reduction in implantation sites and the implantation rate, an interference with the mechanisms of expression of the ER and PR hormone receptors and with their activation pathways. Furthermore, it was observed in EDC-treated mice an altered expression of endometrial receptivity markers, including HOXA 10, LIF, E-cadherin. The results obtained in vivo on mice allow us to assume how fundamental in the fertile life of a woman it is the health workers’ attention and education to the interaction with products that may contain EDC, especially during the preconception period.

## Figures and Tables

**Table 1:** EDCs and reproductive disorders.

Chemicals	Acronym	Exposure	Actions	Authors
**BISPHENOL A**	**BPA**	Plasticizer in food chain (plastics in contact with food), dental sealant, plastic additive	Influence on estrous cycle, affects oocyte maturation, lower serum Estradiol, affect the hypothalamic system, affects morphology and function of oviduct, affect the oocyte and granulosa cells, lower ovarian weight	Tran et al. 2018; Yuang et al. 2019; Muller et al. 2018; Berger et al. 2010; Xiao et al. 2011; Xiaoyan pan et al. 2015; Borman et al. 2015; Crawford et al. 2012; Berger et al. 2008; Jong-Choon et al. 201
**DIBUTYL-PHTALATE**	**DMP**	Plasticizer in polyvinyl, resin solvent, printing inks, paper coating, adhesives, safety glass, cosmetics	decrease in body weight, increase in kidney and liver weight, reduced Hb, RBC and PLT, reduction in T3 and T4 levels, agonists of PXR, effects on steroid hormone biosynthesis	Ema et al. 2000
**MONOBUTYL-PHTALATE**	**MBP**	Plasticizer for nitrocellulose, polyvinyl chloride, lubricant for aerosol valves, skin emollient, hair spray	decrease in body weight, increase in kidney and liver weight, reduced Hb, RBC and PLT, reduction in T3 and T4 levels, agonists of PXR, effects on steroid hormone biosynthesis	Ema et al. 2001
**BIS (2-ETHYLHEXYL)PHTALATE**	**DEHP**	Plasticizer in food chain (plastics in contact with food), deodorants, adhesives, hair spray	decrease in body weight, increase in kidney and liver weight, reduced Hb, RBC and PLT, reduction in T3 and T4 levels, agonists of PXR, effects on steroid hormone biosynthesis	Li et al. 2012
**OP (4-TERT-OCTYLPHENOL)**	**OP**	Detergents, Sanitizers, Defoaming Agents, Agrochemical Emulsifiers, Adhesive, Plastic Industry, Cosmetic and Pharmaceuticals	anemia, leukocytosis, increase in serum cortisol an plasma glucose, uterine calcium transient transport channel antagonist, PR agonist	Tran et al. 2018; Qu et al. 2017
**Chemicals**	**Acronym**	**Exposure**	**Actions**	**Authors**
**PENTACHLOROBIPHENYL**	**PCB 118**	flame retardants, coolants, heat transfer agent	stillbirth, abortion, pregnancy complications, gynecological disease, effects on the development of pinopodes	Qu et al. 2017
**ENDOSULFAN**	**/**	food chain, pesticides	deregulation of ERα, PR, α-SMA, tonic-clonic convulsions, headache, dizziness, ataxia, metabolic disturbances	Milesi et al. 2015; Milesi et al. 2017; Pawar et al. 2015
**MANCOZEB**	**/**	agricultural fungicide, (field crops, fruits, nuts, vegetables, and ornamentals)	contact dermatitis, thyroid hyperplasia, neurotoxicity, suppresion of PGES uterine expression	Aktjar et al. 2020
**CYPERMETHRIN**	**CYP**	insecticide	decrease leveld of estradiol, progesteron, LH, FSH, inhibits expression of PRA in the glandular epithelial cells	Zhou et al. 2017
**TRICLOCARBAN**	**TCC**	antimicrobial agent in soap bars, body washes, plastics, cosmetics	estrogen agonist-Erα, decrease level of FSHR/LH receptors	Costa et al. 2019
**BENZO-A-PYRENE**	**BaP**	cigarette smoke, petroleum products, cahrbroiled foods, contaminated water	estrogen agonist-ERα	Zhao et al. 2014

**Table 2:** EDC and Embryo Implantation Failure.

Authors	Pub. Year	EDC	Used Tech.	Administration	Implantation	PR	ER	Pathway	Animal	Time of Exposure	Condition and Age of Administration
Borman ED et al.	2015	BPA	Immuno-histochemistry	daily injections of 0, 3, 4 or 5 mg BPA in peanut oil	reduction (BPA + stress)	/	/	E-cadherin	Mice (CF-1)	4 days	Adult pregnant female (3–5 months)
Martinez-Pena AA et al.	2019	BPA	Protein extraction/WesternBlot/ELISA	confirmed pregnant females (*n* = 10 females per group) received 0.05 mg/kg/day BPA (previously dissolved in water–ethanol 0.1%) or vehicle via drinking water	reduction	/	/	talin, occudin, E-cadherin	Rats (Wistar)	from GD 6 to lactation day 21	Adult pregnant female
Crawford BR et al.	2012	Triclosan BPA	Anatomic observation	doses of 18 and 27 mg/animal/day on GD 1–3, single doses on GD 2 or 3, combination of triclosan + BPA (4 + 9 mg on GD 1–3)	reduction	/	/	/	Mice (Mus musculus)	3 days	Adult pregnant female (3–6 months)
Berger RG et al.	2008	BPA	Anatomic observation, Enzyme Immunoassay	EXPT 1: females with varied doses of BPA on GD 1–4 (6.75 and 10.125); EXPT 2: inseminated females with a single dose of BPA on GD 0,1, or 2 (6.75 and 10.125)	reduction	/	/	/	Mice (CF-1)	4 days	Adult pregnant female (3–6 months)
Darmani H et al.	2004	BPA dimethacrylate	Anatomic observation	female mice and male mice were exposed to intragastric Bsi-DMA (0,5, 25,and 100 micg/kg) daily for 28 days	reduction	/	/	/	Mice (Swiss Mice)	28 days	Adult female mice
Costa NO et al.	2019	TCC	hematoxylin and eosin	female mice were divided in 4 groups (*n* = 8–11/group): control group; group TCC 0.3 mg/kg; TCC 1.5 mg/kg; TCC 3.0 mg/kg; and treated daily by oral gavage	reduction	/	/	/	Rats (Wistar)	From GD 0—lactational day 21	Adult pregnant female (3 months) effects on female offspring
Akthar I et al.	2020	Mancozeb	Immuno-histochemistry	female mice were administered by oral gavage from GD 1 to GD 8 with doses of Mancozeb (1, 16, and 32 mg/kg BW/day)	reduction	/	/	PGES, COX-2, PGFS, p53	Mice (ICR)	8 days	Adult pregnant female (10–12 weeks)
Pan X et al.	2015	BPA	ELISA, Immuno-histochemistry, Immuno-fluorescence staining	Pregnant females randomly divided into four groups (*n* = 30 for group). From day 0.5 to 4.5 of pregnancy. Daily gavaged with 0, 200, 400, and 600 mg/kg/day BPA in the sesame oil, respectively.	reduced	/	reduction	integrin β3 and trophinin	Mice (Kunming)	5 days	Adult pregnant female (2-month)
**Authors**	**Pub. Year**	**EDC**	**Used Tech.**	**Administration**	**Implantation**	**PR**	**ER**	**Pathway**	**Animal/Study Pop**	**Time of Exposure**	**Condition and Age of Administration**
Pan X et al.	2015	BPA	ELISA, Immuno-histochemistry, Immuno-fluorescence staining	Preg-nant females were randomly divided into five groups (*n* = 30 per group). From 0.5 to 3.5 days in the pregnancy, the pregnant females were daily gavaged with 0, 200, 400, 600, and 800 mg/kg/day of BPA in the sesame oil.	reduced	/	/	/	Mice (Kunming)	5 days	Adult pregnant female (2-month)
Bosquiazzo VL et al.	2009	BPA	RNA extraction & RT/PCR/Immunohistochemistry	s.c. injections of vehicle, BPA (0.05 mg/kg per day or 20 mg/kg per day) on postnatal days 1,3, 5 and 7	/	No affect	reducection	VEGF, ESR1, NCOA3 and NCOR1	Rats (Wistar)	4 days	Newborn Female
Kim JC et al.	2001	BPA	Anatomic observation	Doses of 100, 200, 400, 800 and 1200 mg/kg of BPA (10 mg/kg body weight from GD 1 through 20)	reduction	/	/	/	Rats (Sprague-Dawley)	20 days	Adult pregnant female (10 weeks)
Markey CM et al.	2005	BPA	H & E staining/Morphometric analysis/Immunofluorescence/TUNEL method/	utero exposure to 25 and 250 ng BPA/kg of body weight per day at GD9	/	increase	increase	/	Mice (CD-1)	14 days	Adult pregnant female (8 weeks) effects on female offspring
Tran DN et al.	2018	BPA, OP	RNA extraction/RT PCR/Western blot	female mice from GD 0.5 to GD 3.5 divided into 7 groups (8 mouse each) and given s.c injection of corn oil or ICI (4mg/kg) or estradiol (E2 40micg/kg/day) or BPA (100mg/kg/day) or OP (100 mg/kg/day). Mice in 3 other groups (E2 + ICI, BPA + ICI, OP + ICI) received sc injection of ICI (4mg/kg) 30 min before treatment	reduced(PO); alterated expression(BPA)	reducection	reducection	HOXA10,MUC1, LIF	Mice (ICR)	3 days	Adult pregnant female (8 weeks)
Xiao S. et al.	2011	BPA	Immunohistochemistry	mice were s.c. injected daily with 0, 0.025, 0.5, 10, 40, and 100 mg/kg/day (~ 0, 0.000625, 0.0125, 0.25, 1, 2.5 mg/mouse/day, respectively) of BPA or with 0.01 mg/kg/day E2 (Sigma-Aldrich) in 100 μL sesame oilAldrich) from gestation days 0.5 to 3.5	reducection	/	/		Mice (C57BL6)	3.5 day	Adult pregnant female (2–3 months)
**Authors**	**Pub. Year**	**EDC**	**Used Tech.**	**Administration**	**Implantation**	**PR**	**ER**	**Pathway**	**Animal**	**Time of Exposure**	**Condition and Age of Administration**
LI R et al.	2012	DEHP	SEM/RT—PCR/Immucitochemistry/Western blot	4 groups of 20 mice (control group, 250 mg/kg/day DEHP 500 mg/kg/day DEHP group and 1000 mg/kg/day DEHP group, (10 mice for each group on D5 and D6))	reduction	increase	increase	HOXA10, E-Cadherin, MMp2, p-ERK e NF-KB	Mice (Kunming)	PND22-9GD	Adult pregnant female (8 weeks)
Qu XL et al.	2017	PCB 118	Immunohistochemistry/RT/QPCR Analysis	4 groups with 12 mice in each group	reduction pregnancy rate	No affect	reductiond	Haxa10, ITGB3,DNMT1, DNMT3b	Mice (CD-1)	30 days	Adult pregnant female (8 weeks)
Milesi MM et al.	2017	endosulfan	Immunohistochemistry/image analysis	corn oil, 6 micg/kg/day of endosulfan (Endo6) or 600 mic/kg/day of endosulfan (Endo600) on postnatal days (PND) 1, 3, 5, 7	/	/	increase	MUC1,IGF1,LIF	Rats (Wistar)	4 days	newborn female
Yi T et al.	2018	BaP	RT PCR/Immuhistochemistry/Western Blot/Flow Cytometry/Immunofluorescence	pregnant mice were gavaged with corn oil (control group) or 0.2 mg/kg/day of BaP (treatment group) from GD 1 to GD 6	/	/	/	HOXA10, BMP2, pathways-Wnt, BCL2,BAX	Mice (Kunming)	6 days	Adult pregnant female
Zhao Y et al.	2014	BaP	Plasma sampling and hormone assays/PCR/IHC staining/Western Blot	pregnant mice were dosed with BaP at 0.2, 2 and 20 mg/kg/day from GD1 to GD 5	reduction	reduction	increase	HOXA10,E-Cadherin	Mice (Kunming)	5 days	Adult pregnant female (8 weeks)
Yuan Met al.	2019	BPA	H & E staining and Immunohistochemistry / Cell proliferation assay / Cell culture and treatment / RT-PCR / SDS-PAGE and Immunoblot Analysis / Murine model of oil-induced decidualization	mice were randomly assigned to to control or BPA 4 exposure groups: 0, 1, 10, 100 micg/kg/day dissolved in 10 micl of DMSO and 200 micl of corn oil, and administered by gavage on embryo day 0.5–3.5 and in pseudopregnancy 0.5–3.5 day.	reduction	/	/	SGK1	Mice (ICR)	3 days	Adult pregnant female (7–9 weeks)
Ema M et al.	2001	MPB	radioimmunoassay	administration of DBP-MBuP to pregnant rats and pseudopregnant rats on GD 0 and GD 8 by gastric intubation at 250, 500, 750, or 1000 mg/kg. And pregnancy outcome was determined on day 20 of pregnancy	reduction	/	/	/	Rats (Wistar)	8 days	Adult pregnant female
Martinez-Pe AA et al.	2016	BPA	Immunohistochemistry/Western blot	pregnant wistar dams (F0) received BPA -L (0.05 mg/kg/day), BPA-H (20 mg/kg/day) or vehicle, from GD 6 to 21. F1 females pups were mated at 3 months of age and sacrificed at GD 1, 3, 6, 7.	reduction	/	/	TJ proteins claudin	Rats (Wistar)	6–21 day of lactation	Adult pregnant female
**Authors**	**Pub. Year**	**EDC**	**Used Tech.**	**Administration**	**Implantation**	**PR**	**ER**	**Pathway**	**Animal**	**Time of Exposure**	**Condition and Age of Administration**
Muller JE et al.	2018	BPA	Quantification of uMCs and uNKs/uSAs/High frequency US examination/Measurement of fetal and placental weight/Histology	pregnant female mice exposed to 50 micg/kg/day of BPA or 0.1% ethanol by oral gavage from GD 1 to GD 7	No effect	/	/	/	Mice (C57BL/6)	7 days	Adult pregnant female (8–11 weeks)
Singh et al.	2019	CYP	RNA extraction & RT/PCR/cDNA preparation for quantification gene expression	pregnant rats (F0) were gavaged daily with 0, 1, 10, 25 mg/kg bw/day CYP and 10 micg/kg bw/day Diethylstilbestrol from GD 6 to posnatal day 21	/	increase	increase	HOXA10,a-SMA	Rats (Holtzman)	GD 6-PND 21	Adult pregnant female (9–10 weeks) effects on female offspring
Milesi MM et al.	2015	Endosulfan	Hormone assay/Immunihistochemistry with strepatvidin-biotin preoxidase method/Quantification of cell proliferation and protein expression by image analysis/Dual immunofluorescence staining	received the vehicle 0.2 micg/kg/day of Diethylstilbestrol, 6 micg/kg/day of endosulfan (Endo6) or 600 micg/kg/day of endosulfan (Endo600) on postnatadl days (PND) 1, 3, 5, 7.	reduction	increase	increase	HOXA10, SMRT, SRC-1	Rats (Wistar)	4 days	newborn female
Varayoud J et al.	2011	BPA	Immunihistochemistry/RT and real-time quantitative PCR analysis/Quantification of protein expression	received vehicle BPA.05 (0.05 mg/kg/day), BPA.20 (20 mg/kg/day), DES.2 (0.2 mg/kg/day) or DES.20 (20 mg/kg/day) on PND 1, 3, 5, 7	reduction	reduction	reduction	Hoxa10, ITGB3,EMX-2	Rats (Wistar)	4 days	newborn female
Berger RG et al.	2010	BPA	Immunohistochemical staining/Uterine and ovarian histomorphology/Western Blot	CF-1 mice were injected s.c. with BPA (doses of 0, 3, 3.75, 6.75 and 10.125 mg/animal/day, equivalent to 100, 200, and 300 mg/kg/day) dissolved in 0.45 mll of peanut oil on GD 1–4.	reduction	increase (low dose) reduction (high dose)	increase (low dose) reduction (high dose)	/	Mice (CF-1)	4 days	Adult pregnant female (3–5 months)
Zhou Y et al.	2017	beta-CP	Immunohistochemistry/RT-PCR/Western Blot	40 female mice were assigned to 4 groups of 10 mice each: 1 control group and 3-CP treated groups. The control group (10 mice) was administered with corn oil only, the 3 groups were given corn oil with 5 (10 mice), 10 (10 mice), 20 (mice) mg/kg bw day CP for 3 months through intragastric administration.	reduction	increase	increase	HOXA10	Mice (Kunming)	90 days	Adult female (21 days)
**Authors**	**Pub. Year**	**EDC**	**Used Tech.**	**Administration**	**Implantation**	**PR**	**ER**	**Pathway**	**Animal**	**Time of Exposure**	**Condition and Age of Administration**
Taxvig C et al.	2008	propiconazole, tebuconazole, epoxiconazole and ketoconazole	Hershberger Test/RT-PCR	a total of 50 time-mated rats at GD4 were divided in 5 groups of 10 rats each. The rats were gavaged with vehicle (corn oil) or 50 mg/kg epoxiconazole, 50 mg/kg ketoconazole, 50 mg/kg propiconazole or 50 mg/kg tebuconazole from GD 7 to GD 21 (the dams were given a dosing volume of 2ml/kg body weight	reduction	/	/	/	Rats (Wistar)	14 days	Adult pregnant female
Sapmaz-Metin M et al.	2017	CD	TUNEL assey/Immuno-histochemistry	female BALB/c mice were exposed to 200 ppm Cadmium in their drinking water for either 30 or 60 days	/	/	increase	/	Mice (Balb/c)	Group 2: 30 days; Group 3: 60 days	Adult female
Li Q et al.	2016	BPA	qPCR	4 groups and orally exposed to 0, 60, 600g/kg/d of BPA (designated as BPA-0, BPA-60 , BPA-600, n 8–10/experimental group)	reduction	reduction	no affect	Ihh, Alox15, and Irg1, Hand2 and Hoxa10. if, Fra-1, and Gja1 e MUC1 FGFR/ERK1	Mice (CD-1)	5 weeks	Adult female
Kim HR et al.	2014	BPA	RNA isolation and quantitative RT-PCR analysis (qPCR)/Immunohistochemistry	adult OVX mice were s.c. injected with vehicle (sesame oil 0.1 mL/mouse) or E2 (200 ng/mouse). They were given a single injection of E2 (3-3000 ng), BPA (10-500 mg/kg), P4 (2mg/mouse), GPR30 agonist (1–10 micg), RU486 (1mg/mouse), pretreated with ICI 182,780 (500 micg/mouse) 30 min before.	/	/	increase	Pathway Egr1/2	Mice (ICR)	6 days	Adult female (8 weeks)
Pocar P et al.	2017	DEHP	Histological analysis, reverse-transcription PCR/	gestating F0 mouse dams were exposed to 0, 0.05, 5 mg/kg/day DEHP in the diet from GD 0.5 until the end of lactation	/	/	/	Cdx2, Eomes, Lif	Mice (CD-1)	DPC 0-PND 21	Adult pregnant female effects on female offspring
Ema M et al.	2000	DBP	RNA isolation/Oligonucleotide microarrays/Microarray analysis- data processing/Microarray analysis-identification of significantly atered genes	rats weere given DBP by gastric intubation at 0, 250, 500, 750, 1000, 1250, 1500 mg/kg from GD 0 to GD 8, and the pregnancy outcome was determined on day 20 of pregnancy. The same doses weere given to pseudopregnant rats on GD 0 to GD 8	reduction	/	/	/	Rats (Wistar)	8 days	Adult pregnant female

## Data Availability

Not Applicable.
